# Case report: genetic analysis of a novel frameshift mutation in FMR1 gene in a Chinese family

**DOI:** 10.3389/fgene.2023.1228682

**Published:** 2023-09-07

**Authors:** Chunlei Jin, Xiangdong Zhang, Qiang Lei, Penglong Chen, Hui Hu, Shuangshuang Shen, Jiao Liu, Shixuanbao Ye

**Affiliations:** ^1^ Center of Medical Prenatal Diagnosis, Lishui Maternity and Child Health Care Hospital, Lishui, China; ^2^ Center of Medical Prenatal Diagnosis, Jinhua Maternity and Child Health Care Hospital, Jinhua, China

**Keywords:** FMR1, WES, FMRP, fragile X syndrome, CGG repeat expansion

## Abstract

Fragile X syndrome (FXS) [OMIM 300624] is a common X-linked inherited syndrome with an incidence only second to that of trisomy 21. More than 95% of fragile X syndrome is caused by reduced or absent fragile X intellectual disability protein 1 (FMRP) synthesis due to dynamic mutation expansion of the CGG triplet repeat in the 5′UTR and abnormal methylation of the *FMR1* (fragile X messenger ribonucleoprotein 1) gene [OMIM 309550]. Less than 5% of cases are caused by abnormal function of the FMRP due to point mutations or deletions in the *FMR1* gene. In a proband with clinical suspicion of FXS and no CGG duplication, we found the presence of c.585_586del (p.Lys195AsnfsTer8) in exon 7 of the *FMR1* gene using whole exome sequencing (WES). This variant resulted in frameshift and a premature stop codon after 8 aberrant amino acids. This variant is a novel pathogenic mutation, as determined by pedigree analysis, which has not been reported in any database or literature.

## Introduction

Fragile X syndrome (FXS), an X-linked disorder, is the most common cause of intellectual disability. Repeats of CGG trinucleotides 5 to 40 in the 5′-UTR of the *FMR1* gene exist in the normal population; however, over 200 repeats will lead to the development of the disease. *FMR1* gene amplification, methylation, and transcriptional silencing lead to a loss of FMRP function. In addition, CGG repeats between 55 and 199 also present with the disease, with the most common symptoms being fragile X-related tremor and ataxia syndrome (FXTAS) in men and fragile X primary ovarian hypoplasia (FXPOI) in women. The global mutation rate of FXS patients is 1:5,000 for males and 1:4,000–1:8,000 for females (Santoro et al., 2012; Saldarriaga et al., 2014). Due to incomplete penetrance of the gene, 100% of males with full mutation showed FXS, while females with full mutation have an incidence of approximately one-third due to random inactivation of one X chromosome. Given the high carrier rate of FXS, CGG amplification experiments are often required for patients with intellectual disability. In recent years, with the advent of next-generation sequencing technology, an increased number of mutations within the *FMR1* gene have been discovered. In this study, a novel frameshift mutation was identified in a family with a clinical phenotype suspected of FXS. We recommend that whole exome sequencing (WES) should be the primary consideration in the case of negative CGG amplification tests.

## Materials and methods

### Ethical statement

This study was approved by the Ethics Committee of Lishui Maternity and Child Healthcare Hospital, and all participants gave written informed consent.

### Clinical data

Proband (III:1, Figure 1A): 12 years old, moderate intellectual disability, walking at 17 months, began to talk when 3–4 years old, stuttering, learning difficulties (including counting difficulties), ill-tempered, autism, shyness, eye-gaze avoidance, loves to bite fingers, lack of motor development. Abnormal physical signs: long face, large jaw, large protruding ears, no macroorchidism or obesity. MRI: small patchy abnormal signals in the right thalamus, bilateral lateral ventricles slightly enlarged, small cystic foci in the pineal region. No new phenotypes appeared during 6 months of follow-up.

**FIGURE 1:**
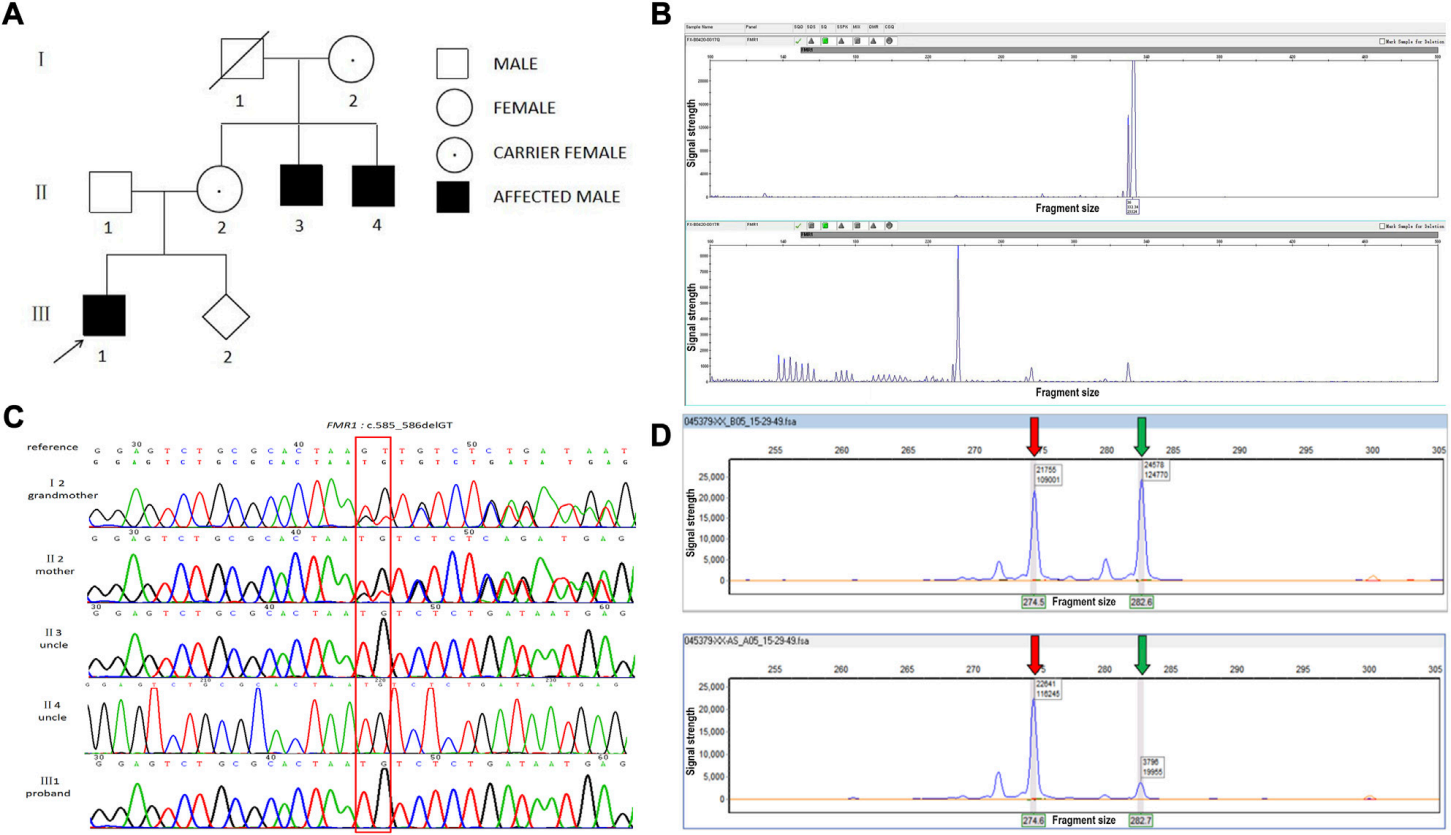
A novel frameshift mutation in the FMR1 gene in a Chinese family. **(A)** FXS pedigree. **(B)** CGG full-length peak of the *FMR1* gene (upper panel) and CGG repeat peak (lower panel). The number of CGG repeats of the *FMR1* gene was calculated by combining this figure. *x*-axis: fragment size, *y*-axis: signal strength. **(C)** Chromatogram of *FMR1*:c.585_586del identified by Sanger sequencing. **(D)** X chromosome inactivation assay using the mother’s DNA. Upper panel: before enzyme digestion; lower panel: after enzyme digestion. The red arrow indicates the X chromosome from the mother and the green arrow indicates the X chromosome from the father. *x*-axis: fragment size, *y*-axis: signal strength.

The proband’s mother (II2, Figure 1A): 33 years old, normal intelligence, illiterate, height 160 cm, weight 53 kg, premature ovarian failure, reproductive history 2-0-1-2, moderate menstrual flow. Abnormal physical signs: long face, hyperextended finger joints.

The proband’s uncle (II3, Figure 1A): 39 years old, height 160 cm, mild intellectual disability, weight 50 kg, illiterate, sweet-tempered, able to communicate normally with people, no stuttering, able to take care of himself. Abnormal physical signs: long face, forehead protrusion, thick lips, large protruding ears.

The proband’s uncle (II:4, Figure 1A): 45 years old, 170 cm, severe intellectual disability that worsened with increasing age, ill-tempered, language impairment, inability to communicate normally with people, no stuttering. The patient had been hospitalized for half a year, had difficulty walking 2 months ago, and had low blood sugar. Abnormal physical signs: forehead protrusion, large protruding ears.

The proband’s grandmother (I2, Figure 1A): 72 years old, normal intelligence, height 160 cm, weight 45 kg, postmenopausal at the age of 50. She gave birth once in her 40s. Abnormal physical signs: long face, hyperextended finger joints.

The clinical data of family members is summarized in Supplementary Table S1.

#### Sample collection (for the core family of the proband)

Five milliliters of peripheral blood was collected from the venous blood of the children and families, of which 2 mL was used to extract genomic DNA using a Qiagen Blood DNA mini kit (Qiagen); Qubit (Qubit ^®^ dsDNA HS Assay Kit, Invitrogen) was used to determine the concentration and the DNA was stored at −20°C until use. At 19 weeks of gestation, amniocentesis was performed under the guidance of ultrasound for prenatal diagnosis.

#### Fragile X syndrome screening test (for the proband and his uncles)

The number of CGG repeats in the 5′UTR of the *FMR1* gene was detected by fluorescence quantitative PCR amplification combined with capillary electrophoresis using a Beijing Reading Gene Fragile X Syndrome Detection Kit, a PCR Instrument, and a Genetic Analyzer (ABI 3500XL). The number of CGG repeats was less than 45 for the normal type, 45 to 54 for the intermediate type, 55 to 200 for the premutation type, and greater than 200 for the full mutation type.

#### Whole exome sequencing (WES) (for the proband)

The test samples were prepared using the Agilent SureSelect method, and the operating manual was followed to complete target region capture and library construction using Agilent Hybrid Capture Reagent. The library was sequenced on the machine after passing quality control analysis using a 2200 Bioanalyzer (Agilent). High-throughput sequencing was accomplished using an Illumina NovaSeq or other throughput system, with a sequencing read length of 2 × 150 bp.

#### Sanger sequencing (for the proband and his parents, uncles, and grandparents)

Sequencing PCR reactions and purification were performed according to the operating procedure of the BigDye^®^ Terminator v3.1 Cycle Sequencing Kit (Applied Biosystems). Ten microliters of Hi-Di (Applied Biosystems) was added to each well, denatured for 5 min, cooled on ice, and transferred to an on-board 96-well plate for sequencing analysis on an ABI 3500XL (Applied Biosystems) platform.

#### X chromosome inactivation detection (for the proband’s mother)

The extracted DNA from the mother was digested with methylation-sensitive endonuclease (HHA I) in one tube, and no endonuclease was added to another other tube as a control, and the parents and other members of the family were subjected to a control experiment. The androgen-receptor (*AR*) gene was amplified by fluorescent labeled primers and then analyzed by capillary electrophoresis and fragment analysis.

## Results

### Detection of CGG repeats of *FMR1* gene

Fluorescence PCR and capillary electrophoresis showed that the proband (III: 1) and his two uncles (II: 3, II: 4) had 36 CGG repeats, which belonged to the normal type (Figure 1B).

### WES and Sanger validation

WES of the proband revealed the presence of c.585_586del (p.Lys195AsnfsTer8) in exon 7 of the *FMR1* gene (NM002024.5). This frameshift mutation results in the conversion of amino acid 195 from lysine to aspartate and causes a premature stop codon. Subsequent sanger sequencing of the family members revealed that the mother and grandmother had c.585_586del heterozygous mutation and that the two uncles presented with c.585_586del hemizygous mutation (Figure 1C).

### X chromosome inactivation assay

The X chromosome inactivation assay was performed on the mother of the proband (II2). The result showed that the ratio of the expressed paternal X chromosome to the expressed maternal X chromosome was approximately 85:15, which was non-random inactivation. The normally expressed *FMR1* gene was expressed to a greater extent than the abnormally expressed *FMR1* gene (Figure 1D).

## Discussion

Fragile X syndrome (FXS) is the most common genetic cause of intellectual disability and is more common in men as an X-linked dominant genetic disorder. Its clinical features include social impairment, autism, delayed language development, neurological dysfunction (seizures and sleep abnormalities), and characteristic appearance (large protruding ears, long faces, highly arched palates, hyperextended finger joints) (Berry-Kravis et al., 2010; Santoro et al., 2012). Although FXS is usually caused by the amplification of the CGG repeat of *FMR1*, a small number of *FMR1* gene mutations leading to FXS have been reported to date (Quartier et al., 2017; Sitzmann et al., 2018; Carroll et al., 2020). These mutations include deletions, splicing, missense, and nonsense mutations.

The *FMR1* gene maps to Xp27.3, has a gene length of 39 kb, and consists of 17 exons and 16 introns. The *FMR1* gene directs the synthesis of FMRP, which is an RNA-binding protein. Dysregulation of the translation/transport/stability of these mRNAs in the absence of FMRP has a cascade effect on many pathways, resulting in a final phenotype of FXS (Maurin et al., 2014). This protein is found in many tissues, including the brain, testes, and ovaries. In the brain, it is thought to be able to regulate specific gene expression during neurodevelopment and affect the plasticity of neural cell synapses, resulting in the inability of dendrites to develop to mature size and shape (Bassell, 2011; Deng and Klyachko, 2021; Prieto et al., 2021).

In this study, based on the proband’s clinical presentation and pedigree analysis, the diagnosis was speculated to be FXS, which was found to be negative after performing routine CGG repeat amplification tests. Subsequent WES of this family revealed the presence of c. 585_586del in exon 7 of the *FMR1* gene, which may form a truncated ion form of FMRP. Quartier et al. (2017) reported that deletion of exon 17 of *FMR1* leads to a truncated FMRP and a decreased expression level of this truncated form of FMRP in the patients. Therefore, we propose that c. 585_586del in exon 7 in this study may also lead to a decreased expression level of the truncated form of FMRP and contribute to the phenotypes (Quartier et al., 2017).

Male carriers of mutations in the *FMR1* gene are generally FXS patients, while female carriers have high clinical heterogeneity, and phenotypes from normal intelligence to severe intellectual disability may exist, which are more likely to be associated with learning disabilities, socio-emotional, and mental health problems (Hagerman et al., 2017; Reches, 2019). During the development of female embryos, most genes on one X chromosome in each cell are randomly inactivated, resulting in approximately half of the cells in the body expressing genes from each X chromosome. The theoretical ratio of two types of cells is 50:50. The type of X chromosome inactivation in the female population (the ratio of X inactivated cells in the father to X inactivated cells in the mother) follows the normal distribution, and a small number of normal women will deviate from 50:50. Usually, a ratio greater than or equal to 90:10 is used as the criterion for determining non-random inactivation of X; however, 80:20 is also used as a criterion for non-random inactivation (Robinson et al., 2001; Uehara et al., 2001). It is believed that only a few embryonic progenitor cells will continue to form the brain; therefore, it is believed that the ratio of affected X-activated cells to silenced cells significantly affects the expression level of FMRP in the developing central nervous system, and the difference in this ratio may lead to significant changes in the phenotype of FXS women. In fact, the phenotype is commonly milder in females than in males (Loesch et al., 2004; Bartholomay et al., 2019).

The males of this family have some clinical features, such as intellectual disability, prominent forehead, flaring ears, and long face. The mother and grandmother of the proband only showed excessive extension of long face and fingers, which may be related to the skewness of the female X chromosome. In this study, the X chromosome inactivation test was performed on the mother of the proband, and the result showed that the ratio of X chromosome from the father to X chromosome from the mother was approximately 85:15, which was non-random inactivation. The normal expression of the *FMR1* gene was greater than the abnormal expression, which can be used to explain the slight clinical phenotype of female carriers.

At present, conventional detection methods for FXS mainly use PCR and capillary technology to detect the presence of CGG trinucleotide amplification in the 5′UTR of the *FMR1* gene. The application of chromosome microarray technology to detect *FMR1* gene deletion (full, partial, or mosaicism) has also been reported (Coffee et al., 2009; Gonçalves et al., 2016; Ciaccio et al., 2017). WES is recommended for *FMR1* point mutation detection in cases with negative CGG tests or *FMR1* gene deletion when FXS syndrome is suspected. According to HGMD (professional 2023.2), 89 pathogenic mutations (45 gross deletions, 16 repeat variations, 7 gross insertions, 5 splicing, 4 missense, 1 nonsense, 3 regulatory, 4 small deletions, 3 insertions/indels, and 1 complex rearrangement) have been reported. Among them, at least 17 pathogenic mutations (5 splicing, 4 missense, 1 nonsense, 4 small deletions, and 3 insertions/indels), accounting for 19.1% of all the reported pathogenic mutations, could be identified by WES. The proband in this study had a negative CGG expansion result; however, WES identified a frameshift mutation, which was verified by Sanger sequencing. This frameshift mutation disrupts the reading frame of the *FMR1* gene, resulting in a premature stop codon after 8 aberrant amino acids and forming a truncated protein. Therefore, the proband and his two uncles developed an FXS phenotype. Population databases showing *FMR1* gene variant c.585_586del are very rare and it has not been reported in the gnomAD, ExAC, HGMD, or ClinVar database or in relevant literature.

In summary, a c.585_586del frameshift mutation in the *FMR1* gene was detected in the proband of this family by WES, which was the cause of FXS in the proband. This study provides a theoretical basis for genetic counseling and fertility guidance in this family. Moreover, the discovery of a new variant also expands the mutation spectrum of this gene. We also emphasize the importance of using WES as a clinical tool. The discovery of *FMR1* gene mutations may lead to a better understanding of the function of FMRP, especially at the synapse, and an understanding of the pathways underlying these alterations is essential to develop many targeted therapies for FXS. Understanding the relationship between specific mutations and the different effects of FMRP on pre-synaptic and/or post-synaptic function will be interesting and should be further studied in clinical cases and animal models (Hagerman et al., 2014; Jacquemont et al., 2014; Sitzmann et al., 2018; Prieto et al., 2021).

## Data Availability

The original contributions presented in the study are included in the article/Supplementary Material, further inquiries can be directed to the corresponding authors.
