# Supplementary material for: Case report: genetic analysis of a novel frameshift mutation in FMR1 gene in a Chinese family
Source: Front Genet. 2023 Sep 7;14:1228682. doi: 10.3389/fgene.2023.1228682 (PMC10512415; doi:10.3389/fgene.2023.1228682)
Supplement: Supplementary file 1 [file Table1.DOCX]

| Clinical  Family members | **I-2** (Grandma) | **II-2（**Mother**）** | **II-3（**Uncle**）** | **II-4（**Uncle**）** | **III-1（**Proband**）** |
| --- | --- | --- | --- | --- | --- |
| Age | 72 | 33 | 45 | 39 | 12 |
| Gender | Female | Female | Male | Male | Male |
| Special facies (long face, prominent forehead, ear) | Long face | Long face | Yes | Yes | Yes |
| Joint hyperextension | Yes | Yes | No | No | No |
| Speech disorder | No | No | Yes | No | Yes |
| Intellectual disability | No | No | Yes (Severe) | Yes (Mild) | Yes (moderate) |
| Autism | No | No | Yes | No | Yes |
| Delayed or impaired motor development | No | No | Yes | No | Yes |
